# Antitumor Activity of Carboxymethyl Pachymaran with Different Molecular Weights Based on Immunomodulatory and Gut Microbiota

**DOI:** 10.3390/nu15214527

**Published:** 2023-10-25

**Authors:** Yalin Zhao, Xi Feng, Lijia Zhang, Wen Huang, Ying Liu

**Affiliations:** 1 College of Food Science and Technology, Huazhong Agricultural University, Wuhan 430070, China; yalinzhao@webmail.hzau.edu.cn (Y.Z.); 13297021290@163.com (L.Z.); huangwen@mail.hzau.edu.cn (W.H.); 2 Department of Nutrition, Food Science and Packaging, San Jose State University, San Jose, CA 95192, USA; xi.feng@sjsu.edu

**Keywords:** carboxymethyl pachymaran, molecular weight, structural characterization, antitumor activities, gut microbiota

## Abstract

Carboxymethyl pachymaran (CMP) was treated via high-temperature and cellulase hydrolysis to obtain HTCMP, HTEC-24, and HTEC-48. The chemical structure and in vivo antitumor activities of the four types of CMPs were investigated. Compared with CMP (787.9 kDa), the molecular weights of HTCMP, HTEC-24, and HTEC-48 were decreased to 429.8, 129.9, and 68.6 kDa, respectively. The viscosities and particle sizes of the CMPs could also decrease with the decline in the molecular weights. All the CMPs showed antitumor abilities, but HTEC-24 exhibited the best activity. In the animal study, when curing the spleen and thymus, CMPs displayed immunomodulatory effects by increasing the secretion of IFN-γ and IL2 in mice. The CMPs also exerted an antitumor ability by regulating the gut microbiota in tumor-bearing mice. Our results established a foundation to develop an antitumor drug with CMP.

## 1. Introduction

Polysaccharides have caught attention due to their immunomodulatory activity, non-toxicity, and other advantages. The molecular weight of polysaccharides greatly affects their physicochemical properties and biological activities. Some polysaccharides with a large molecular weight tend to have poor water solubility and a complex structure, which makes it difficult for them to pass though cell membranes to exert biological activities [1]. Thus, it is a hot research topic to obtain polysaccharides with a smaller molecular weight with a similar functionality. The active sites of polysaccharide chains are usually several polysaccharide fragments or oligosaccharides on the chains [2]. Therefore, the degradation of polysaccharides is the main means to obtain highly active polysaccharide fragments or oligosaccharides with low molecular weights, good water solubilities, and simple structures. The common methods used to obtain low-molecular-weight polysaccharides are chemical degradation, physical degradation, and biodegradation [3]. Chemical degradation is the process of hydrolyzing polysaccharides using acids or oxides. Physical degradation is the process of reducing the molecular weight using ultrasounds, homogenization, irradiation, etc. The biodegradation of polysaccharides uses specific enzymes. Enzymes can directly break chemical bonds with a high efficiency. Moreover, enzymatic reactions are carried out under specific mild conditions [4,5]. However, the overdegradation of polysaccharides can lead to the destruction of the structure and make degradation products less active [6].

*Poria cocos* (Schw.) Wolf is the dried sclerotium of the fungus *P. cocos*, which has been used as a medicine in China for a long time, and is also an important dual-use resource for medicine and food [7,8]. Polysaccharides are the main components of *P. cocos*, accounting for more than 90% of its dry weight, and they are also one of the main active ingredients [9]. Pachymaran has a variety of physiological activities, such as immunomodulatory [10], antitumor [11], antioxidant [12], and anti-inflammation effects [7]. However, more than 90% of pachymaran is an alkali-soluble polysaccharide, with a poor water solubility and low activity, which greatly limits the use of pachymaran in clinics [13]. Therefore, various modifications have been applied to improve its water solubility and biological activity. Studies have shown that the chemical modification of *P. cocos* polysaccharides can alter their biological activities [14]. The most common modification is carboxymethylation, which can change the water solubility and biological activities [15,16,17].

Numerous research data have indicated that carboxymethyl pachymaran (CMP) has the effect of inhibiting the growth of tumor cells (HepG2, S180, HT29, MCF-7, etc.) in vitro, and CMP can also improve the immunity of mice through immune regulation and exert a certain antitumor effect [18,19,20]. However, the inhibitory effect is greatly affected by the preparation method and structure of CMP. Many studies have explored the mechanisms of the antitumor effects of CMP from different perspectives, such as apoptosis, tumor invasion and migration, the immune escape of tumors, etc. [18,21], but fewer studies have investigated the antitumor effects of different molecular weights of CMP from the immune modulation and gut microbiota.

The aims of this study were to obtain degraded CMP through high-temperature and cellulase hydrolysis treatments and explore the structural changes based on their molecular weights, apparent viscosity measurement, particle size distribution assay (PSD), and Fourier transform infrared spectra assay (FT-IR). The effect of degradation treatments on the tumor growth in H22 tumor-bearing mice was investigated via immunomodulation and gut microbiota.

## 2. Materials and Methods

### 2.1. Materials and Reagents

CMP (purity > 85.0%, carboxymethyl substitution degree: 0.691) was purchased from Wuhan Runge Biotechnology Co., Ltd. (Wuhan, China). Cellulase (BR, 50 u/mg) and CTX were purchased from Shanghai Yuanye Biotechnology Co., Ltd. (Shanghai, China). RPMI-1640 basic medium and fetal bovine serum (FBS) were from Gibco Company, Carlsbad, CA, USA. Other reagents were of an analytical grade.

### 2.2. Degradation Treatments of CMP

The CMP solution (20 mg/mL) was prepared with pure water, autoclaved for 20 min at 121 °C and 0.2 MPa, and freeze-dried to obtain samples labeled as HTCMP [22]. The method of enzymatic hydrolysis was based on that of Fu et al. with some modifications [23]. The pH of the HTCMP solution was adjusted to 4.5, and cellulase was then added to the sample solution (enzyme dosage: 0.5% (*w*/*w*), calculated by the mass of CMP). Then, the enzymatic reaction was carried out at 40 °C. The hydrolyzed samples were collected at 24 h and 48 h, respectively. Lastly, the hydrolyzed samples were placed in boiling water for 10 min to inactivate the cellulases. The degraded polysaccharides were concentrated and freeze-dried to obtain samples. The CMP treated with high temperature and enzymatic hydrolysis for 24 h was labeled as HTEC-24. The CMP treated with high temperature and enzymatic hydrolysis for 48 h was labeled as HTEC-24. CMP, HTCMP, HTEC-24, and HTEC-48 were collectively referred to as CMPs.

### 2.3. Molecular Weight Determination

The parameters of the molecular weights of the CMPs were determined using SEC-MALLS-RI with reference to the method of Morris et al. [24,25]. The weight average molar mass (M_w_), number average molar mass (M_n_), polydispersity index (M_w_/M_n_), and radius of rotation (R_z_) were detected using a DAWN HELEOS-II multi-angle laser scatter meter (He-Ne laser, λ = 663.7 nm, Wyatt Technology Co., Goleta, CA, USA).

A 0.1 mol/L NaCl solution was prepared as the mobile phase, then it was filtered with a 0.22 µm filter membrane and degassed for 15 min. The sample solutions of 1 mg/mL were prepared using the mobile phase solution and centrifuged (8000 rpm, 15 min). The supernatant was collected and filtered through a 0.45 µm membrane. The test parameters were a sample volume of 0.1 mL, mobile phase flow rate of 0.4 mL/min, sample solution refractive index d_n_/d_c_ of 0.138 mL/g, and column temperature of 25 °C.

### 2.4. Apparent Viscosity Analysis

The apparent viscosity of the CMPs was determined using a AR-2000ex rheometer form Waters Technology Co., (Milford, MA, USA). A preparation of 5 mg/mL CMP solution was made using distilled water. The diameter and gap of the plates were 40 mm and 0.5 mm. The viscosity was determined at a shear rate between 0 and 100 s^−1^ using the frequency scanning mode of data acquisition. The results were plotted with the viscosity as the vertical coordinate and the shear rate as the horizontal coordinate.

### 2.5. Particle Size Distribution (PSD)

The particle size distribution of the CMPs was measured using a laser particle size distribution meter (Mastersizer 2000, Malvern, London, UK) with a method modified from Alvarez-Sabatel et al. [26]. The CMPs were fully dispersed in distilled water and added to the sample cell. The solution of the samples was stirred at 1000 rpm.

### 2.6. Fourier Transform Infrared Spectra (FT-IR) Assay

The dried sample was mixed with KBr powder in a certain ratio (1:100) and ground into a non-reflective powder. The clear flakes were pressed using a tablet press and then scanned using the Nexus 470 FT-IR spectrometer (Thermo Nicolet, Waltham, MA, USA) at wavelengths from 4000 to 400 cm^−1^. The KBr flakes were used as a control [27].

### 2.7. Antitumor Activity In Vivo

#### 2.7.1. H22 Cell Culture

H22 cells were purchased from the Wuhan Procell Life Science & Technology Co., Ltd. (Wuhan, China) and cultivated in RPMI-1640 basic medium supplemented with 10% *v*/*v* FBS and 1% *v*/*v* antibiotics (containing 100 U/mL penicillin and 100 μg/mL streptomycin). H22 cells were incubated under standard culture conditions (37 °C, 5% CO_2_).

#### 2.7.2. Animals and Experimental Design

A total of 140 male specific-pathogen-free (SPF) KM mice (20.0 ± 2.0 g, 4–6 weeks old) were purchased from the Laboratory Animal Centre of Huazhong Agricultural University (Wuhan, China, certificate number: SYXK(Ei)2020-0084). All mice were grown under the following conditions: a room temperature of 24–25 °C, a relative humidity of 70–75%, a 12 h light/12 h dark cycle, and free access to water.

The animal experiment was conducted with the approval of the Committee of Animal Experimental Ethical Inspection of the Laboratory Animal Centre, Huazhong Agriculture University (approved number: HZUMO-2023-0060).

After 7 days of acclimatization, 130 mice were injected with 0.2 mL of H22 cells adjusted to 2 × 10^6^ cells/mL subcutaneously in the right axillary. Normal mice (A) did not receive any treatments. The H22-bearing mice were randomly divided into 13 groups with 10 mice per group, as follows: model control (B) group, positive control (CTX, C) group, low-dose CMP (CMP-L, D1), middle-dose CMP (CMP-M, D2), high-dose CMP (CMP-H, D3), low-dose HTCMP (HTCMP-L, E1), middle-dose HTCMP (HTCMP-M, E2), high-dose HTCMP (HTCMP-H, E3), low-dose HTEC-24 (HTEC-24-L, F1), high-dose HTEC-24 (HTEC-24-H, F2), low-dose HTEC-48 (HTEC-48-L, G1), high-dose HTEC-48 (HTEC-48-H, G2), and CMP + CTX (H) groups.

When tumor cells were inoculated for 24 h, the mice of group C were intraperitoneally injected with 30 mg/kg/d of CTX for 14 days. The mice of groups D1, D2, and D3 were administered 100, 200, and 300 mg/kg/d of CMP; the mice of groups E1, E2, and E3 were administered 100, 200, and 300 mg/kg/d of HTCMP; and the mice of groups F1, F2, G1, and G2 were administered 50 and 200 mg/kg/d of HTEC-24 and 50 and 200 mg/kg/d of HTEC-48 for 14 days. The mice of group H were administered 200 mg/kg/d of CMP and intraperitoneally injected with 30 mg/kg/d of CTX for 14 days. During the experiments, the weights of the mice were recorded daily. After the last administration, the mice were fasting for 12 h, but they could drink water freely. All mice were sacrificed via cervical dislocation after the removal of the eyeballs for blood. The blood samples of each group were collected in heparinized tubes. The tumor, spleen, and thymus were cut and weighed. The collected tissues and serum were stored at −80 °C for further analysis.

#### 2.7.3. The Inhibition Rate

The antitumor activity of the CMPs was expressed as an inhibition ratio calculated as follows:

$$Inhibition\;rate\;\left(\%\right)=\frac{W_{A}-W_{B}}{W_{A}}\times 100$$

Here, *W_A_* was the average tumor weights of group A and *W_B_* was the average tumor weights of experimental groups, respectively.

#### 2.7.4. Determination of Organ Index

All mice had their weights recorded before being sacrificed. The thymus and spleen were surgically excised, removed, and weighed. The immune organ index was calculated with the following formula:

$$Spleen\;or\;thymus\;Index=\frac{spleen\;or\;thymus\;weight\;\left(\mathrm{mg}\right)}{body\;weight\;\left(g\right)}$$

#### 2.7.5. H&E Assays

The thymus, spleen, and tumor were fixed with 4% paraformaldehyde. After paraffin embedding, sectioning, and staining with hematoxylin and eosin (H&E), the metallographs were taken at 200× magnification using an inverted microscope to observe the H&E staining morphologic changes of the immune organs and tumors.

#### 2.7.6. Measurement of Serum Immunoglobulin and Cytokines

The blood samples from all mice were centrifuged (3000 rpm, 10 min, 4 °C), and the serum was collected. The mouse serum was stored at −80 °C. The levels of IFN-γ, IL-2, and IgG in the serum were determined using ELISA kits (Wuhan Fengbin Technology Co., Ltd., Wuhan, China).

#### 2.7.7. Gut Microbiota Analysis

The 16S rRNA Illumina sequencing technique was applied to analyze the difference between the gut microbiota of mice in groups A, B, and F1. DNA was extracted from mouse cecum then quantified by Nanodrop, and the quality of DNA extraction was examined via 1.2% agarose gel electrophoresis. The obtained DNA was used as a template for PCR amplification, using the forward primer 5′-ACTCCTACGGGAGGCAGCAG-3′ and reverse primer 5′-GGACTACHVGCCTWTCTAAT-3′. The PCR amplification products were quantified via fluorescence, and, after passing the test, the libraries were built and sequenced using the TruSeq Nano DNA LT Library Prep Kit of the Illumina platform.

#### 2.7.8. Statistical Analysis

All of the experimental results were expressed as mean ± standard deviation (SD). A one-way ANOVA was performed using SPSS 26.0 statistical analysis software (IBM, Armonk, NY, USA), and a Tukey’s test was used for analysis of variance (*p* < 0.05). Plots were made using Origin 2018 software (Origin Lab Corporation, Northampton, MA, USA).

## 3. Results and Discussion

### 3.1. Structural Characterization of CMPs

#### 3.1.1. Molecular Weights of CMPs

The molecular weight of polysaccharides is closely related to their biological activity and physical properties. The relative molar mass of CMPs was determined using the SEC-MALLS-RI system [28]. The M_w_, M_n_, M_w_/M_n_, and R_z_ values of the CMPs are shown in Table 1. The weight average molar mass and M_w_/M_n_ values of CMP, HTCMP, HTEC-24, and HTEC-48 were 706.4 (2.479), 429.8 (2.186), 129.9 (1.713), and 68.62 kDa (1.507), respectively. It can be seen that high-temperature treatment and cellulase hydrolysis treatment could reduce the molecular weights of CMP effectively.

A high temperature can accelerate the breaking of chemical bonds and degradation of the compounds. However, a temperature below 140 °C will only specifically destroy the glycosidic bonds in the polysaccharide molecules and will not affect the substituents on the chains of polysaccharides [29]. In addition, cellulase enzymes can directly break the β-1,4 glycosidic bond [30]. Hu et al. [31] hydrolyzed the mulberry leaf polysaccharide (MLP) to obtain MLO (MLP with different molecular weight) using hemi-cellulase, which reduced its molecular weight from 51.21 kDa to 852.63 Da. MLO has a more uniform molecular weight distribution.

#### 3.1.2. The Apparent Viscosity of CMPs

**Figure 1:**
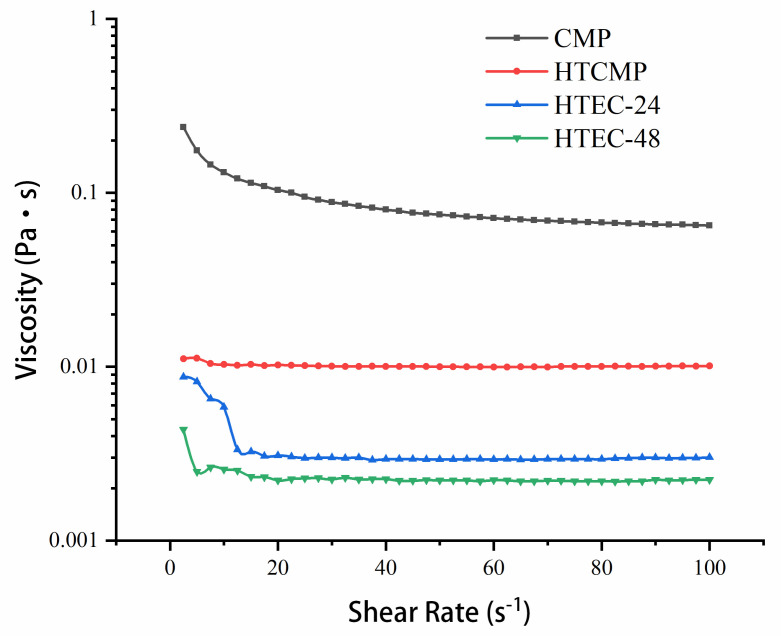
The rheological properties of CMP, HTCMP, HTEC-24 and HTEC-48.

The viscosity of polysaccharide is affected by the solution temperature, molecular weight, length and conformation of polysaccharide chains, etc. [32]. The apparent viscosity of the CMPs was determined using a rheometer, and the results are shown in Figure 1. When the shear rate was fixed, the viscosities of HTCMP, HTEC-24, and HTEC-48 were 13.6%, 4.0%, and 3.0% of CMP, respectively. The high-temperature treatment and enzymatic hydrolysis treatment reduced the viscosity of the CMP solutions effectively.

Furthermore, the viscosities of the CMPs decreased significantly as the shear rate increased from 5 to 20 s^−1^. It is similar to the results reported after the enzymatic digestion of mulberry leaf polysaccharides [31]. Polysaccharides’ shear dilution is associated with the disruption of intermolecular hydrogen bonds [32]. It is a typical characteristic of non-Newtonian fluids [33,34]. The decrease in the viscosity of CMP after high-temperature treatment and enzymatic treatment may be due to the decrease in the molecular weight of the polysaccharides, the shortening, and the change in the conformation of polysaccharide chains [29].

#### 3.1.3. The Particle Size Distribution

**Figure 2:**
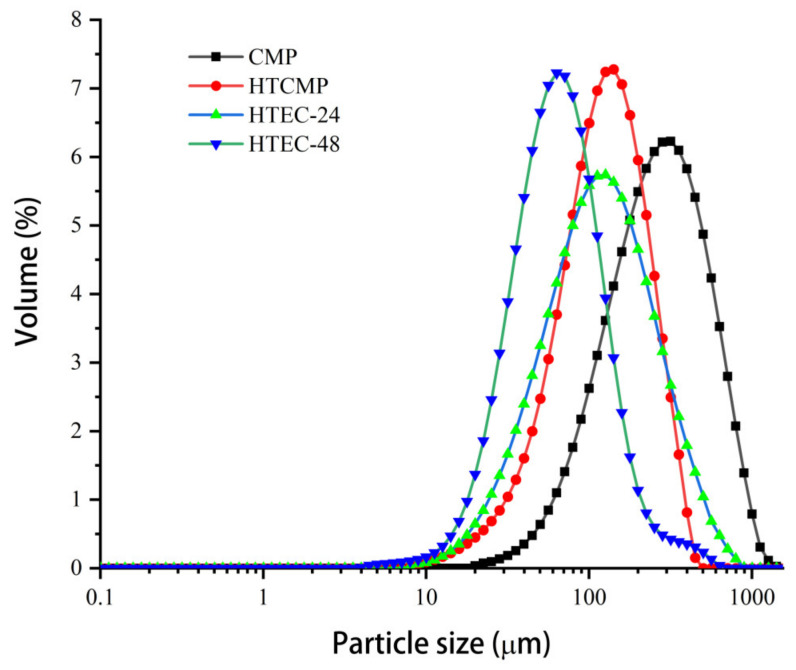
The particle size distribution of CMP, HTCMP, HTEC-24 and HTEC-48.

The particle size distribution of the CMPs was determined using a laser particle size distribution meter and expressed as a percentage by volume. The results are shown in Table 2 and Figure 2. Small and spherical particles were measured by D values [2,3], but D values [3,4] are more sensitive to large, irregularly shaped particles, such as clusters and aggregates [35]. The trend observed here suggests that high temperatures significantly reduce the average particle size of CMP (*p* < 0.05). Enzymatic hydrolysis and high-temperature treatment could both reduce the average particle size of polysaccharides. The reduction was more significant with the prolongation of the enzymatic hydrolysis time (*p* < 0.05). The four samples showed a single peak distribution. The particle size distributions of the degradation-treated polysaccharides were narrower than the CMP, indicating that the high-temperature and enzymatic treatment can reduce the presence of large molecules in the CMP and make the polysaccharides more homogeneous in size [26]. The results were consistent with the results of the molecular weight and viscosity measurements described previously. The decreasing molecular weights of HTCMP, HTEC-24, and HTEC-48 were reflected in their decreasing viscosities, narrower particle size distributions, and smaller particle sizes. In addition, HTEC-24 and HTEC-48 had lower molecular weights and viscosities than HTCMP, indicating that high-temperature treatment and cellulase hydrolysis treatment can break different bonds in the CMP and degrade the polysaccharides.

#### 3.1.4. FT-IR Spectra of CMPs

**Figure 3:**
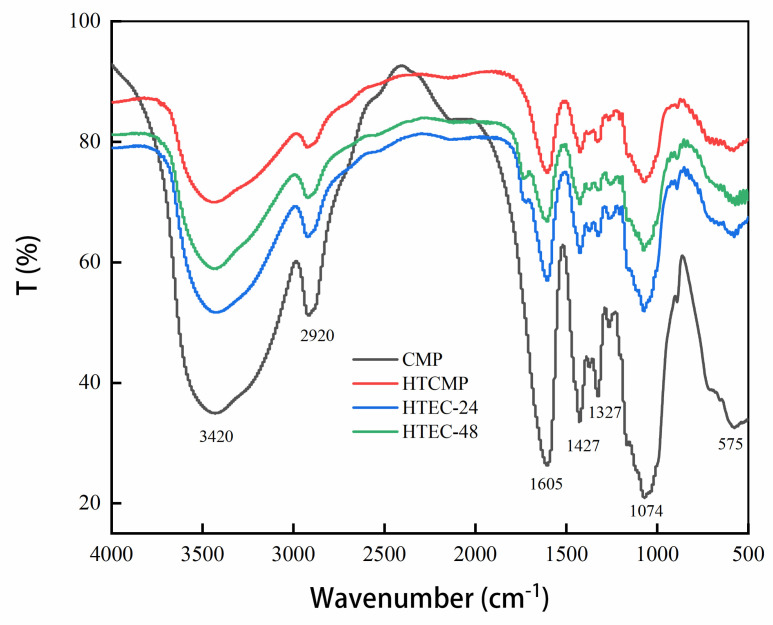
FT-IR spectra of CMP, HTCMP, HTEC-24 and HTEC-48.

The FT-IR spectra of the CMPs are shown in Figure 3. The absorption peaks at 3420 cm^−1^ and 2920 cm^−1^ are generated by O-H and C-H stretching vibrations in CMPs [27], respectively. The absorption peak at 1605 cm^−1^ was generated by the asymmetric absorption vibration of C=O, the absorption peak at 1427 cm^−1^ was generated by the vibration of C-H of -CH_3_COOH [10], and the absorption peak at 1327 cm^−1^ was caused by the vibration of C=O. These are characteristic absorption peaks for carboxymethyl [19].

The high-temperature treatment at 121 °C did not affect the substituents on the chains of polysaccharides while destroying glycosidic and non-covalent bonds. Moreover, cellulase only hydrolyzes the β-1,4 glycosidic bonds. Therefore, the basic structures of the polysaccharide chains of the CMPs were not very different. This was consistent with the results of Figure 3. Similar results were found by Ma et al. [36]. They obtained the degraded polysaccharides from sweet corncob and found that the basic structure did not change and only the polysaccharides chains were slightly altered. In conclusion, the degradation methods of high-temperature treatment and cellulase hydrolysis did not affect the primary functional group status of CMP.

### 3.2. Effects of CMPs in H22 Tumor-Bearing Mice

#### 3.2.1. Effect of CMPs on Tumor Inhibition Rate in Tumor-Bearing Mice

**Figure 4:**
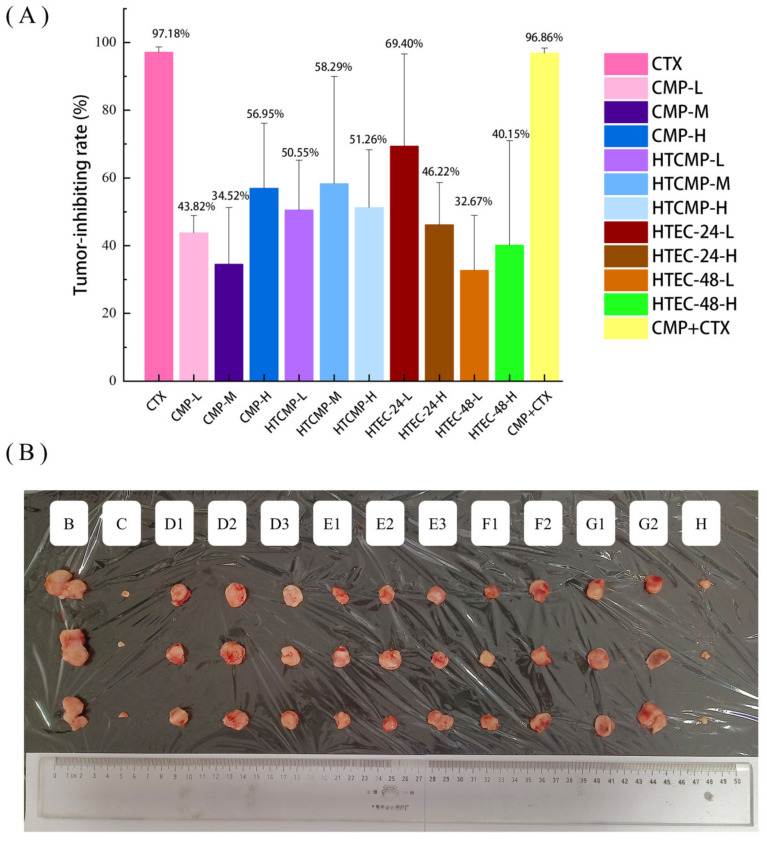
(**A**) The tumor inhibition rates of different treatment groups (*n* = 10). (**B**) The tumors of mice.

A solid tumor model was established through the subcutaneous injection of H22 tumor cells under the right axilla of mice, so that the effect of CMPs on hepatocellular carcinoma in mice could be evaluated. During the experiment, two mice died, and the survival rate of the mice was 90%. The body weights of the mice are revealed in Table 3. After 14 days of administration treatment, the weight increments of mice in groups C and H were significantly lower than those of Groups A and B (*p* < 0.05), indicating that CTX not only inhibited tumor growth but also affected the mice in a negative way. We found that the mice groups B and C showed signs of weakness, poor appetite, and reduced activity, while no significant adverse effects occurred in the CMP-treated groups. The tumor inhibition rates of different groups are shown in Figure 4A. All the CMP groups showed certain inhibitory effects on tumors, but the inhibitory effects varied depending on the molecular weights of the CMPs. The tumor inhibition rates of HTCMP were higher than those of CMP at both low and medium doses. Compared with groups D2 and E2, group F1 was administered at a lower dose but showed a higher tumor inhibition rate (69.41%), probably because enzymatic hydrolysis exposed the active groups on the polysaccharide chains and improved the antitumor activity of CMP.

In studies on the antitumor effects of various polysaccharides on H22 tumor-bearing mice, most of their tumor inhibition rates were in the range of 20–50%, such as 39.13% for Hericium erinaceus polysaccharide [37], 44.7% for polysaccharide from Gastrodia Elata [38], 37.05% for polysaccharides from the root of Angelica sinensis [39], and 29.48% for lily polysaccharides [40]. The higher tumor inhibition rate and significant antitumor effect of HTEC-24 may be attributed to the fact that hydrolysis reduced the molecular weight of HTEC-24, which made it easier for it to be absorbed in the bodies of mice. Numerous studies have shown that when the molecular weight of polysaccharides was reduced within a certain range their antioxidant activity [41,42,43], antitumor activity [44,45], hypoglycemic activity, and immunomodulatory activity were enhanced. The tumor inhibition rate of group G1 was lower than that of group F1, probably because enzymatic hydrolysis was excessive, which damaged the active groups of polysaccharide chains.

#### 3.2.2. Effect of CMPs on Organ Indexes in Tumor-Bearing Mice

**Figure 5:**
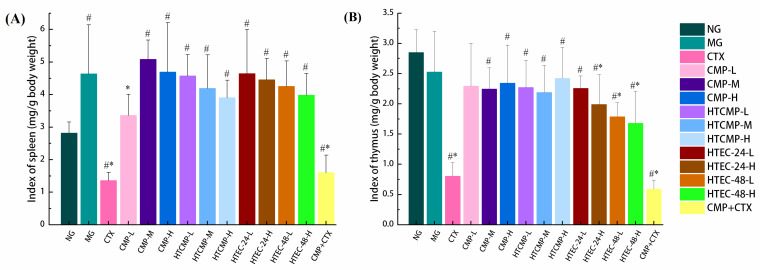
The indexes of the spleen (**A**) and thymus (**B**). # *p* < 0.05 compared to group A. * *p* < 0.05 compared to group B.

The spleen and thymus are important organs in the immune system of the body and play a role in the link between humoral and cellular immunity [46]. Based on the masses of the thymus and spleen, the thymus index and spleen index can be calculated, which can reflect the immunotoxicity of antitumor drugs and the immune status of the body. The results are shown in Figure 5. The spleen index was significantly higher in group B (4.64 mg/g) than in group A (2.82 mg/g) (*p* < 0.05), indicating that the mice in group B may have entered the stage of pathological splenomegaly and the non-specific immune function of the mice may be impaired [10]. The spleen index was significantly lower in group C (1.36 mg/g) compared to group B, indicating that the CTX produced severe damage to the spleen of the mice (*p* < 0.05). Compared with group C, the spleen index was higher in group H (1.60 mg/g), indicating that CMP had a certain restorative effect on the spleen of the mice. Low doses of CMP (100 mg/kg) brought the spleen index in mice close to normal. As shown in Figure 5B, the thymus indexes of all mice of the CMP-treated groups were significantly higher compared to group C (*p* < 0.05), which indicated that CMP had a protective effect on the thymus of tumor-bearing mice.

#### 3.2.3. H&E Staining

**Figure 6:**
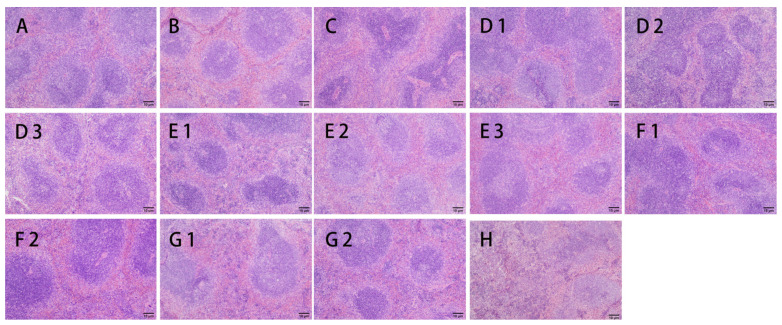
Effects of CMP, HTCMP, HTEC-24 and HTEC-48 on the morphology of the spleens of mice (HE, 200×) ((**A**) normal group; (**B**) model group; (**C**) positive control group (CTX); (**D1**) low-dose CMP group, 100 mg/kg/d; (**D2**) middle-dose CMP group, 200 mg/kg/d; (**D3**) high-dose CMP group, 300 mg/kg/d; (**E1**) low-dose HTCMP group, 100 mg/kg/d; (**E2**) middle-dose HTCMP group, 200 mg/kg/d; (**E3**) high-dose HTCMP group, 300 mg/kg/d; (**F1**) low-dose HTEC-24 group, 50 mg/kg/d; (**F2**) high-dose HTEC-24 group, 200 mg/kg/d; (**G1**) low-dose HTEC-48 group, 50 mg/kg/d; (**G2**) high-dose HTEC-48 group, 200 mg/kg/d; (**H**) CMP + CTX group).

**Figure 7:**
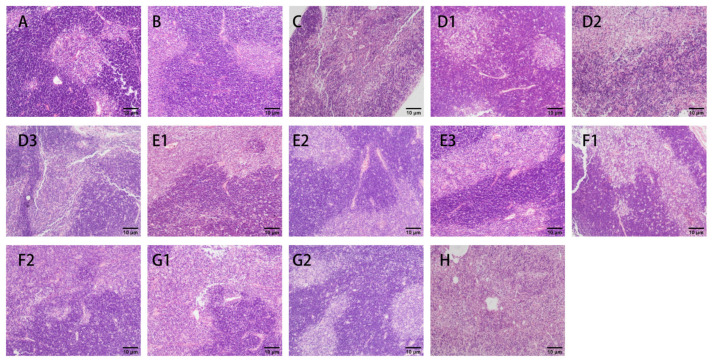
Effects of CMP, HTCMP, HTEC-24 and HTEC-48 on the morphology of the thymuses of mice (HE, 200×) ((**A**) normal group; (**B**) model group; (**C**) positive control group (CTX); (**D1**) low-dose CMP group, 100 mg/kg/d; (**D2**) middle-dose CMP group, 200 mg/kg/d; (**D3**) high-dose CMP group, 300 mg/kg/d; (**E1**) low-dose HTCMP group, 100 mg/kg/d; (**E2**) middle-dose HTCMP group, 200 mg/kg/d; (**E3**) high-dose HTCMP group, 300 mg/kg/d; (**F1**) low-dose HTEC-24 group, 50 mg/kg/d; (**F2**) high-dose HTEC-24 group, 200 mg/kg/d; (**G1**) low-dose HTEC-48 group, 50 mg/kg/d; (**G2**) high-dose HTEC-48 group, 200 mg/kg/d; (**H**) CMP + CTX group).

**Figure 8:**
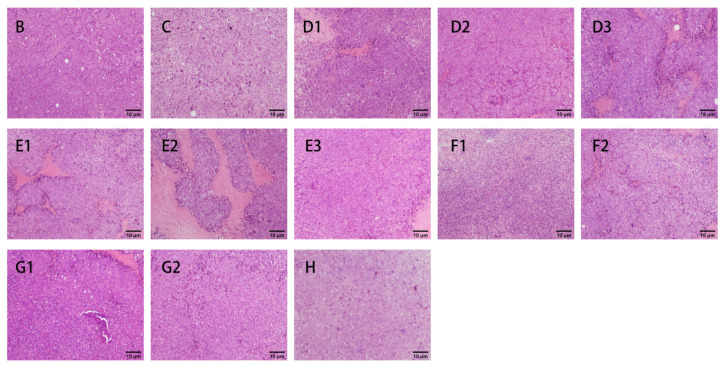
Effects of CMP, HTCMP, HTEC-24 and HTEC-48 on the morphology of the tumor cells (HE, 200×) ((**B**) model group; (**C**) positive control group (CTX); (**D1**) low-dose CMP group, 100 mg/kg/d; (**D2**) middle-dose CMP group, 200 mg/kg/d; (**D3**) high-dose CMP group, 300 mg/kg/d; (**E1**) low-dose HTCMP group, 100 mg/kg/d; (**E2**) middle-dose HTCMP group, 200 mg/kg/d; (**E3**) high-dose HTCMP group, 300 mg/kg/d; (**F1**) low-dose HTEC-24 group, 50 mg/kg/d; (**F2**) high-dose HTEC-24 group, 200 mg/kg/d; (**G1**) low-dose HTEC-48 group, 50 mg/kg/d; (**G2**) high-dose HTEC-48 group, 200 mg/kg/d; (**H**) CMP + CTX group).

Hematoxylin and eosin staining (H&E staining) is a common means of histological analysis in which the nuclei and cytoplasm of cells within animal tissues are stained with different dyes to blue-purple and red colors, respectively [47]. The observations of the spleen, thymus, and tumor H&E staining in each group of mice at 200× are shown in Figure 6, Figure 7 and Figure 8.

As shown in Figure 6, the dark areas are the white pulp, and the light areas are the red pulp. In group A, the boundary between the red and white pulp of the spleen was well defined, and the splenic lymphocytes were closely arranged and evenly distributed. Compared with group A, group B had a blurred boundary between the white and red pulp, with the red pulp of the spleen becoming larger and the white pulp of the spleen becoming smaller. Splenocyte lysis could be observed, and the spleen was loosened. The elevated splenic index in group B was due to the destruction of immune cells, and the spleen was in the stage of pathological enlargement [10]. In group C, the percentage of white pulp of the spleen was reduced, and it was irregular in shape. The central artery was enlarged, and the splenocytes were irregular, indicating that the CTX had caused some damage to spleen cells. In the CMP-treated group, the degree of intertissue cell loosening in the spleen of mice declined, and the proportion of white pulp of the spleen increased, gradually recovering to the original structure of the spleen. Group F1 was most similar to group A.

As shown in Figure 7, the dark areas are the cortex, and the light areas are the medulla. The boundary of the cortices and medullas of the thymuses in group A was clear; the cortex was thicker, and the lymphocytes were densely arranged. Compared with group A, group B had blurred boundaries between the cortices and the medullas, with reduced proportions of medullas. In group C, there was no clear boundary between the cortices and the medullas, and vacuoles appeared, with the proportions of cortices greatly reduced, indicating the severe destruction of thymic tissues.

As shown in Figure 8, the tumor cells in group B grew well and were tightly arranged. The cell borders were clear, and the apoptosis of tumor cells was rarely observed. In contrast, group C showed loosely arranged tumor cells with lamellar necrosis, had increased apoptotic cells, and appeared pinker in color [47]. Apoptosis was observed to varying degrees in all the groups administered CMPs, with the most pronounced apoptosis in group F1.

The results indicated that CMPs may enhance the immune effect of mice to a certain extent by acting on the spleen and thymus and increase the antitumor ability of the body.

#### 3.2.4. Analysis of Serum Indicators and Cytokine Assays

**Figure 9:**
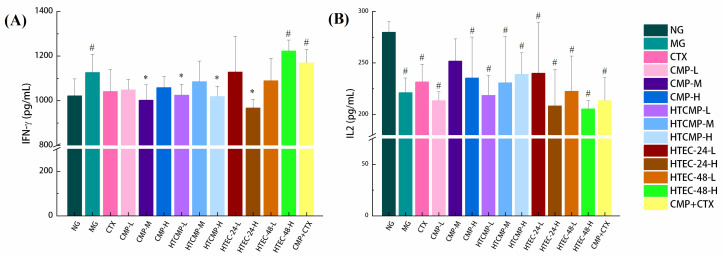
Effects of CMP, HTCMP, HTEC-24 and HTEC-48 on cytokine IFN-γ (**A**) and IL2 (**B**) in serum of mice. The data were expressed as means ± SD (*n* = 6). ^#^ *p* < 0.05 compared to group A. * *p* < 0.05 compared to group B.

Helper T cells (Th’s) can secrete cytokines to regulate the proliferation and differentiation of B lymphocytes and induce the expression of receptors in the immune response of the organism [48]. Therefore, the level of cytokines in the serum can reflect the changes in the immune function of the body. Tumor necrosis factor (TNF-α), interleukins (IL-1β, IL-2, IL-6, etc.), and interferon (IFN-γ) are considered common cytokines. The levels of immunizing factors in the serum of each group of mice are shown in Figure 9. Compared with group A, the IL-2 level in group B was reduced significantly; the IFN-γ levels were slightly increased but not significantly different (*p* > 0.05). We speculate that the growth of tumors stimulated the bodies of mice, which led to a slight increase in the level of IFN-γ.

Different treatment groups varied in regards to cytokines: Compared with group B, TNF-α levels were increased significantly (*p* < 0.05) in the medium-dose CMP group (200 mg/kg). Group F1 (HTEC-24-L) showed the highest tumor inhibition rate, but its effect on cytokines was not significantly different. In a study by Wang et al. [49], LNT significantly elevated serum levels of TNF-α and IL2 in H22 tumor-bearing mice. The non-significant effect of CMPs on the elevation of cytokines in serum in the present study may be related to the method of establishing the tumor-bearing mice model.

#### 3.2.5. Effect of CMPs on Immunoglobulin Content in Serum of Tumor-Bearing Mice

**Figure 10:**
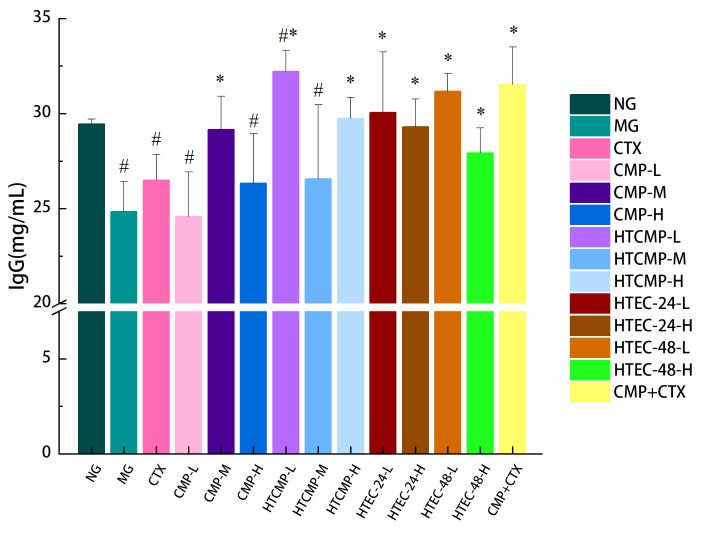
Effects of CMP, HTCMP, HTEC-24 and HTEC-48 on immunoglobulins IgG in serum of mice. The data were expressed as means ± SD (*n* = 6). ^#^ *p* < 0.05 compared to group A. * *p* < 0.05 compared to group B.

Immunoglobulins are a class of glycoproteins found in blood and tissue fluids, which are produced by plasma cells generated by the proliferation and differentiation of B lymphocytes following antigenic stimulation [48]. The level of immunoglobulins in serum is often used to reflect the immune function of body. Ig-A, Ig-G, and Ig-E are categorized as common antibodies. IgG is the most abundant immunoglobulin in serum, which is the most important material basis of immune responses [50]. The levels of immunoglobulin in the serum of each group of mice are shown in Figure 10. IgG levels were significantly lower in group B (*p* < 0.05), indicating the humoral immunity was reduced. Compared with group A, the IgG levels of groups treated with CMP-M, HTCMP-H, HTEC-24-L, and HTEC-24-H were similar, and the IgG levels of groups treated with HTCMP-L and HTEC-48-L were increased but not significantly different. The above results suggested that HTCMP and HTEC-48 can enhance humoral immunity in mice by increasing IgG levels in serum.

### 3.3. Effect of HTEC-24 on Gut Microbiota of Tumor-Bearing Mice

**Figure 11:**
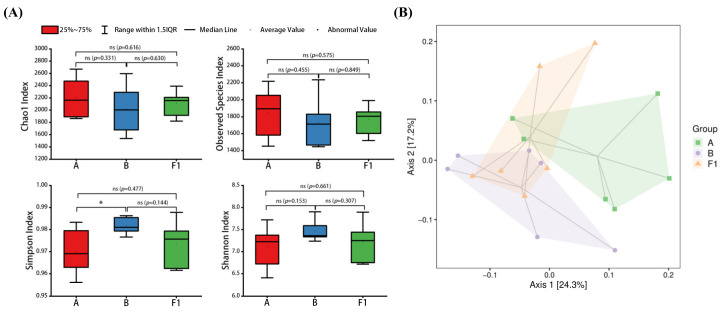
Analysis of gut flora richness and diversity in different treatment groups: (**A**) alpha diversity evaluated from Chao1, observed species, Shannon, and Simpson indexes; (**B**) weighted UniFrac principal coordinate analysis (PCoA) based on OTU abundance (*n* = 6). * *p* < 0.05.

Alpha diversity is a measurement of species richness, diversity, and evenness in a localized homogeneous habitat, also known as intrahabitat diversity, which typically includes the Chao1 index, observed species index, Shannon diversity index, Simpson diversity index, and so on. The Chao1 and observed species indexes are both related to the richness of the community, with larger values representing higher richness. Smaller values of the Simpson index and larger values of the Shannon index indicate a richer diversity of the gut microbiota. The Chao1, observed species, Simpson, and Shannon indexes were similar in groups A, B, and F1, indicating that there was no significant difference in the abundance and diversity of gut microorganisms (Figure 11A). However, there was a slight decrease in the Chao1 and observed species indexes in group B compared with group A, suggesting that the tumors exerted a slight effect on the abundance of the gut microbiota of the mice. The Chao1 and observed species indexes of group F1 were slightly higher than those of group B, and the Simpson index was reduced, suggesting HTEC-24 had a restorative effect on the gut microbiota of mice.

PCoA analysis can visualize the interrelationships between samples using the distance between samples on a two-dimensional plot. With Bray–Curtis distance, it can be observed that group A and group B were farther away from each other, and group F1 became closer to group A (Figure 11B). This indicated that the tumors negatively affected the gut microbiota environment of the mice, and HTEC-24 can improve the gut microbiota environment of the mice.

**Figure 12:**
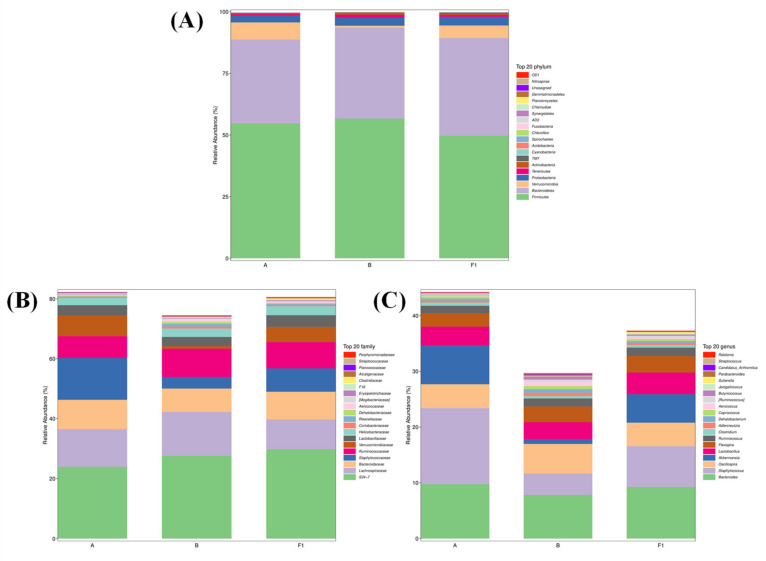
Effect of HTEC-24 on the composition of gut microbiota: (**A**) relative abundance (%) of the microbial community at the phylum level in different treatment groups (*n* = 6 per group); (**B**) relative abundance (%) of the microbial community at the family level in different treatment groups (*n* = 6 per group); (**C**) relative abundance (%) of the microbial community at the genus level in different treatment groups (*n* = 6 per group).

To further investigate the different taxonomic compositions of groups of gut flora, the distribution of the species composition was compared at the phylum, family, and genus taxonomic levels. At the phylum level, we found that the dominant flora of intestinal microorganisms in all groups of mice mainly consisted of Firmicutes, Bacteroidetes, and Verrucomicrobia. The relative abundance distribution of Verrucomicrobia decreased from 6.96% to 0.84% in group B, but its relative abundance distribution increased to 5.11% in group F1 (Figure 12A), suggesting that the distribution of gut microbiota was somewhat disturbed by the tumors of the mice, and the HTEC-24 was beneficial in restoring the homeostasis of gut microbiota. At the family level, the dominant flora in all groups of mice were mainly S24-7, Lachnospiraceae, Bacteroidaceae, Staphylococcaceae, Ruminococcaceae, and Verrucomicrobiaceae. The relative abundance of Staphylococcaceae in group B was reduced from 13.97% to 3.86% (Figure 12B), and the relative abundance of Verrucomicrobiaceae was reduced from 6.96% to 0.85%, but the relative abundance distributions of all of them were improved and almost restored to the normal level after HTEC-24 administration treatment. At the genus level, the dominant flora in all groups of mice were mainly Bacteroides, Staphylococcus, Oscillospira, Akkermansia, and Lactobacillus. Compared with group A, the relative abundance in group B of Staphylococcus and Akkermansia decreased from 0.116% and 6.956% to 0.062% and 0.846%, respectively. The relative abundance of Staphylococcus was elevated (0.131%) after HTEC-24 treatment. The relative abundance of Akkermansia was increased to near normal levels (5.113%) (Figure 12C).

Many studies have shown that chemotherapy drugs cause intestinal disturbances [51,52]. Cai et al. investigated the effect of oyster polysaccharides (CHP) on S-180 tumor-bearing mice and found that the ratio of the Firmicutes to Bacteroidetes ratio decreased significantly (1.14 to 0.99) [53]. After treatment with 5-FU and CHP, the abundance of Verrucomicrobia (phylum level) and Akkermansia (genus level) increased significantly. In line with our findings, the abundance of Verrucomicrobia and Akkermansia was higher in group F1 after treatment with HTEC-24. Akkermansia is a potential probiotic that improves the metabolic profile and mucus layer thickness. It has a protective effect on the intestinal tract. In summary, HTEC-24 may exert antitumor effects by modulating the abundance of gut microbiota in tumor-bearing mice.

Because the gut microbiota is rich in carbohydrate-active enzymes that can hydrolyze polysaccharides, the digestion and absorption of polysaccharides in the body occurs primarily in the intestines. The polysaccharides can be degraded by gut microbes into short-chain organic acids, including acetic acid, propionic acid, and butyric acid, which can maintain the epithelial barrier function, regulate epithelial cell proliferation, and modulate immune responses [10]. The intestinal microflora promotes lymphocyte development and immune function and also regulates the immune function in the immune system [54]. We speculate that HTEC-24, with a suitable molecular weight size, is more easily absorbed and utilized by the bodies of mice. In addition, HTEC-24 may promote the secretion of immune factors by regulating the gut microbiota and increasing the abundance of beneficial bacteria to exert a better antitumor effect in mice.

## 4. Conclusions

In this study, CMPs with different molecular weights were obtained using high-temperature and cellulase hydrolysis treatments. The molecular weights, chemical structures, physicochemical properties, and in vivo antitumor activities of different CMPs were evaluated. Both high-temperature treatments and enzymatic hydrolysis could degrade CMP by destroying the bonds of polysaccharide chains. In addition, CMPs had certain antitumor effects, but HTEC-24 showed higher in vivo antitumor activity. CMPs could increase the serum levels of IFN-γ and IL-2, act on the spleens and thymuses of mice, and improve the immune activity of the tumor-bearing mice. Moreover, HTEC-24 could improve the composition of gut microbiota and increase the abundance of beneficial bacteria in tumor-bearing mice and had a restorative effect on gut microbiota disorders in tumor-bearing mice. This study provides a theoretical foundation for antitumor research on CMP. In future studies, it may be necessary to further investigate the antitumor mechanism of CMP using molecular mechanisms.

## Figures and Tables

**Table 1:** Molecular parameters of CMP, HTCMP, HTEC-24 and HTEC-48 (means ± SD).

Sample	M_w_ (kDa)	M_n_ (kDa)	M_w_/M_n_	R_z_ (nm)
CMP	706.4 ± 38.4	285.0 ± 28.4	2.479 ± 0.281	137.5 ± 2.2
HTCMP	429.8 ± 14.9	196.7 ± 10.2	2.186 ± 0.136	72.7 ± 2.1
HTEC-24	129.9 ± 5.1	75.86 ± 3.61	1.713 ± 0.106	49.8 ± 3.9
HTEC-48	68.62 ± 3.15	45.53 ± 3.82	1.507 ± 0.144	29.9 ± 5.9

**Table 2:** The average particle sizes for the CMP, HTCMP, HTEC-24 and HTEC-48.

Sample	d (0.5)	D (3,2) (μm)	D (4,3) (μm)
CMP	251.479 ± 3.459 ^a^	180.884 ± 0.918 ^a^	300.084 ± 9.227 ^a^
HTCMP	116.367 ± 4.444 ^b^	83.976 ± 3.007 ^b^	131.413 ± 5.317 ^b^
HTEC-24	109.186 ± 7.333 ^b^	76.433 ± 3.470 ^c^	141.536 ± 13.866 ^b^
HTEC-48	60.215 ± 2.159 ^c^	47.384 ± 0.805 ^d^	75.746 ± 7.878 ^c^

^a, b, c, d^ Different superscripts in the same column indicate significant differences (*p* < 0.05).

**Table 3:** Effects of CMP, HTCMP, HTEC-24 and HTEC-48 on the body weight of the mice.

	Initial Weight	Final Weight	Weight Increment
A	31.52 ± 2.72	37.48 ± 3.67	5.96 ± 1.83
B	31.45 ± 4.01	38.85 ± 4.66	7.40 ± 1.63
C	31.59 ± 2.53	34.65 ± 2.27 *	3.06 ± 1.89 ^#^*
D1	31.24 ± 2.65	37.96 ± 2.87	6.71 ± 1.66
D2	31.86 ± 1.90	38.72 ± 2.36	6.86 ± 1.88
D3	31.86 ± 2.45	38.52 ± 2.57	6.66 ± 0.89
E1	31.84 ± 1.93	38.19 ± 2.55	6.35 ± 2.03
E2	31.70 ± 1.94	37.89 ± 3.01	6.19 ± 1.76
E3	31.69 ± 2.16	39.43 ± 3.19	7.74 ± 1.98
F1	31.54 ± 1.78	38.30 ± 3.01	6.76 ± 2.40
F2	31.64 ± 1.51	38.48 ± 2.18	6.84 ± 2.32
G1	31.55 ± 1.21	37.86 ± 1.68	6.31 ± 1.64
G2	31.59 ± 1.74	39.51 ± 3.23	7.92 ± 1.75
H	30.92 ± 1.76	33.50 ± 2.47 ^#^*	2.58 ± 1.51 ^#^*

^#^ *p* < 0.05 compared to group A. * *p* < 0.05 compared to group B.

## Data Availability

Not applicable.
